# Functional and biological insights of rCollinein-1, a recombinant serine protease from *Crotalus durissus collilineatus*

**DOI:** 10.1590/1678-9199-JVATITD-1471-18

**Published:** 2019-04-08

**Authors:** Johara Boldrini-França, Ernesto Lopes Pinheiro-Junior, Eliane Candiani Arantes

**Affiliations:** 1 School of Pharmaceutical Sciences of Ribeirão Preto - FCFRP/USP, Ribeirão Preto, São Paulo, Brazil.; 2 Graduate Program in Ecosystem Ecology, University of Vila Velha, Av. Comissário José Dantas de Melo, 21, Boa Vista II, 29102-920, Vila Velha, ES, Brazil.

**Keywords:** snake venom, serine proteases, thrombin-like enzymes, coagulation

## Abstract

**Background:**

The prevalent class of snake venom serine proteases (SVSP) in Viperidae venoms is the thrombin-like enzymes, which, similarly to human thrombin, convert fibrinogen into insoluble fibrin monomers. However, thrombin-like serine proteases differ from thrombin by being unable to activate factor XIII, thus leading to the formation of loose clots and fibrinogen consumption. We report the functional and biological characterization of a recombinant thrombin-like serine protease from *Crotalus durissus collilineatus*, named rCollinein-1.

**Methods:**

Heterologous expression of rCollinein-1 was performed in *Pichia pastoris* system according to a previously standardized protocol, with some modifications. rCollinein-1 was purified from the culture medium by a combination of three chromatographic steps. The recombinant toxin was tested *in vitro* for its thrombolytic activity and in mice for its edematogenicity, blood incoagulability and effect on plasma proteins.

**Results:**

When tested for the ability to induce mouse paw edema, rCollinein-1 demonstrated low edematogenic effect, indicating little involvement of this enzyme in the inflammatory processes resulting from ophidian accidents. The rCollinein-1 did not degrade blood clots *in vitro*, which suggests that this toxin lacks fibrinolytic activity and is not able to directly or indirectly activate the fibrinolytic system. The minimal dose of rCollinein-1 that turns the blood incoagulable in experimental mice is 7.5 mg/kg. The toxin also led to a significant increase in activated partial thromboplastin time at the dose of 1 mg/kg in the animals. Other parameters such as plasma fibrinogen concentration and prothrombin time were not significantly affected by treatment with rCollinein-1 at this dose. The toxin was also able to alter plasma proteins in mouse after 3 h of injection at a dose of 1 mg/kg, leading to a decrease in the intensity of beta zone and an increase in gamma zone in agarose gel electrophoresis

**Conclusion:**

These results suggest that the recombinant enzyme has no potential as a thrombolytic agent but can be applied in the prevention of thrombus formation in some pathological processes and as molecular tools in studies related to hemostasis.

## Background

Proteases are present in the venom of most snake families and are structurally classified as metalloproteases and serine proteases. Snake venom serine proteases (SVSP) are widely found in venoms from Viperidae and Crotalidae snakes. SVSPs act primarily on plasma proteins, generating a variety of physiological effects on platelet aggregation, blood coagulation, fibrinolysis, blood pressure, as well as complement and immune systems [1-7].

The SVSP-coding genes belong to the trypsin/kallikrein family and comprise five exons and four introns, from which exons 2 to 5 encodes the mature SVSP [8-10]. SVSPs are synthesized in the form of a zymogen of about 260 amino acids, containing a signal peptide and a propeptide of 18 and 6 amino acid residues, respectively. Mature SVSPs are generally single chain glycoproteins exhibiting six disulfide bridges, with some exceptions, such as *Cerastes cerastes* RP-34 toxin [11], brevinase from *Agkistrodon blomhoffii brevicaudus* [12] and DAnase from *Deinagkistrodon acutus* [13], which present an extra unpaired cysteine that confers them a disulfide-linked dimeric form.

Alike chymotrypsin, trypsin and thrombin, the catalytic domain of SVSPs contains the triad His57, Asp102 and Ser195 that catalyzes the peptide bond cleavage by an acylation and deacylation reaction, in which the serine has a nucleophilic role and the histidine acts as a proton donor/acceptor [14, 15]. SVSPs enzymatic activity is inhibited by a variety of synthetic and natural compounds [16], especially those that modify the reactive serine, such as PMSF (phenylmethylsulfonyl fluoride) [17].

Snake venom thrombin-like serine proteases (SVTLEs) are the prevalent class of serine proteases from Viperidae venoms. SVTLEs have similar activity to that of human thrombin by cleaving fibrinopeptides A and/or B from fibrinogen, leading to the formation of fibrin monomers. However, these enzymes differ from thrombin in many ways. One of the most relevant differences of thrombin-like enzymes when compared to thrombin is their inability in activating factor XIII of coagulation, which forms crosslinks between fibrin monomers. Thus, a loose clot is formed, leading to fibrinogen depletion. The consumption coagulopathy caused by thrombin-like serine proteases may, secondarily, activate the fibrinolytic system in response to the formation of soluble fibrin monomers. Tissue plasminogen activator (tPA) binds to a positively charged lysine in the fibrin surface, and the resulting fibrin-tPA complex cleaves plasminogen into plasmin, activating the fibrinolytic system and removing the loose clot [18, 19].

By mimicking some natural regulatory components of coagulation, SVSPs may have applications in the treatment and diagnostics of certain hemostatic disorders, which have raised interest in basic and applied researches related to these toxins [20]. A classic example of a SVSP that has been used for therapeutic purposes is Batroxobin (Defibrase^®^, Pentapharm - CH), a thrombin-like enzyme isolated from *Bothrops atrox* venom, indicated to treat patients with deep vein thrombosis, myocardial infarction, angina, ischemia, among other clinical applications [21-24]. Gyroxin from *Crotalus durissus terrificus* venom is another example of a SVTLE that has been used as a fibrin sealant for therapeutic purposes [25, 26]. Several preclinical studies and clinical trials have been conducted, in which this enzyme was used as scaffold for stem cells in tissue repair [27-30], in cicatrization of chronic venous ulcers [31, 32] and in the regeneration of peripheral nervous system [33-38].

Despite the great therapeutic and biotechnological potential of thrombin-like enzymes, the mechanisms by which these toxins affect blood clotting and other physiologic systems were not fully understood yet. In general, SVSPs are multifunctional toxins that exhibit a wide range in substrate specificity and can thus act on several prey or victim systems, such as platelet aggregation, blood coagulation, fibrinolysis, blood pressure, complement and nervous system [39]. Therefore, it is important to extensively explore the functionality of this toxin class to seek for new therapeutic and biotechnological applications.

Collinein-1 is a 29.5 kDa thrombin-like serine protease isoform from *C. d. collilineatus* venom that cleaves preferentially the Aα chain of fibrinogen. The recombinant form of collinein-1 (rCollinein-1) was previously obtained with functional integrity from *P. pastoris* heterologous system [40]. Therefore, in this work we investigate the *in vivo* effects of rCollinein-1, as well as its fibrinolytic properties, aiming to deepen the functional knowledge regarding this recombinant toxin, enabling its possible therapeutic applications.

## Methods

### **Heterologous expression and purification of rCollinein-1 in *Pichia pastoris***

The heterologous expression of rCollinein-1 was based on the previously reported method [40], with some modifications. For large-scale expression, *P. pastoris* cells transformed with the recombinant plasmid were pre-inoculated into a 125 mL Erlenmeyer flask containing 10 mL of BMGY medium and incubated at 30 ºC under constant stirring of 210 rpm. After 24 hours, the culture was inoculated into a 2 L Erlenmeyer flask containing 500 mL of BMGY medium and incubated at 30 ºC under constant stirring of 210 rpm until an optical density of 2 to 6 at 600 nm. After reaching the desired optical density, the culture was centrifuged at 1500 *xg*, the supernatant was discarded, and the cells were resuspended in 100 mL of BMMY medium, pH 6.0, in a 1 L Erlenmeyer flask at 26 ºC under constant stirring of 210 rpm. Methanol was replaced at a final concentration of 0.75% every 24 h for induction of protein expression. After 96 h of induction, the culture was centrifuged at 2500 *xg*, the supernatant was separated, filtered and used for purification of the recombinant protein. The recombinant enzyme was purified from the culture medium by a combination of three chromatographic steps. The culture medium was firstly fractionated by immobilized metal affinity chromatography (IMAC) using a Ni^2+^-Agarose resin (Ni-NTA Agarose, Qiagen, Hilden - DE) at gravitational flow. Elution of the recombinant protein was performed with a segmented gradient from 10 mM to 250 mM imidazole. The fractions containing rCollinein-1 were then submitted to a second chromatographic step using a weak cation exchange column (CMC-52, 20 cm x 4 cm), accomplished in a fast protein liquid chromatography system (FPLC) Äkta Purifier UPC10 (GE Healthcare, Chicago, Illinois - USA). Elution was carried out using a discontinuous gradient of the equilibrium buffer (50 mM sodium acetate buffer, pH 5.0) up to 1 M. The absorbance was monitored at 280 nm. Finally, the purity of the recombinant protein was confirmed by a strong ion exchange chromatography in a Mini S 4.6/50 PE (GE Healthcare, Chicago, IL - USA) column, using the same buffer of the second chromatographic step. The percentage of protein recovery was calculated by the Unicorn^®^ 5.2 program (Amersham, Little Chalfont - UK). The fractions eluted from the column were analyzed in a 13.5% polyacrylamide gel electrophoresis (SDS-PAGE), dialyzed against ultrapure water, lyophilized and stored at -20 °C until use.

### Mass spectrometry

A solution containing the recombinant protein was co-sedimented with the matrix 2,5-dihydroxybenzoic acid (DHB - 10 mg/mL in solution containing 0.2% formic acid and 80% acetonitrile), in a ratio of 1:1 (v/v). The samples were ionized by MALDI (Matrix-assisted laser desorption/ionization) and analyzed in the Ultraflex II mass spectrometer (MALDI-TOF/TOF) (Bruker Daltonics, Billerica, MA - USA). The laser power was adjusted to 32%, with an incidence of 5,000 to 7,000 shots to generate a satisfactory signal-to-noise ratio. Ions were detected with the analyzer operated in the positive linear mode.

### Thrombolytic activity

The thrombolytic activity of rCollinein-1 was evaluated on blood clots formed *in vitro*, as described by Toni [41]. For this, 500 μL of fresh human blood (collected from healthy volunteers without anticoagulant addition) was added to 24-well plates, followed by incubation for 1 h at room temperature for clot formation. After this period, the clots were incubated for 24 to 48 hours at 37 °C with different doses of the enzyme (25, 50, 100 μg in 500 μL of saline). Samples containing only saline and 25 μg of Actilyase (Boehringer Ingelheim, Ingelheim am Rhein - DE) were incubated with the clot under the same conditions as negative and positive control, respectively. After incubation, thrombolytic activity was estimated based on the weight of the remaining thrombus and the results were expressed in milligrams. The assays were performed in a series of three replicates, and the data were adjusted with the respective standard errors using GraphPad Prism software, version 5.0 (GraphPad Software Inc., San Diego, CA - USA). The experiments were made in accordance with the principles of Research Ethics Committee under the license number 334.

### ***In vivo* assays**

### Animals

Male Swiss mice were obtained from the biotherium of the School of Pharmaceutical Sciences of Ribeirão Preto (FCFRP/USP). The animals were kept in an environment with controlled temperature (23 ± 1 °C) and humidity (55 ± 5 %) with a light/dark cycle of 12 h. Food and water were provided ad libitum. Mouse experimental models are in accordance with the Ethical Principles in Animal Experimentation under the license number 2012.1.414.53.4.

### Edematogenic Activity

Edematogenic activity was evaluated in Swiss mice (18-22 g). The animals were divided into groups containing three animals each, which received different doses of rCollinein-1 (10, 25 and 50 μg) diluted in 50 μL of sterile saline (0.15 M NaCl) by subcutaneous injection in the subplantar region of the left hind paw. The right hind paw received only saline as a negative control. The thickness of both paws was measured using plethysmometer (model 7140, Ugo Basile, Gemonio, VA - IT) at different time intervals (30, 60, 120, 240 and 480 min). The results were calculated by the difference between the values ​​obtained for both paws and expressed in percentage paw thickness increase in relation to the initial measurements.

### Blood incoagulability

Male Swiss mice (18-22 g) were separated into eight groups (n = 3), which received different doses of the enzyme (0.025, 0.05, 0.1, 0.5, 1.0, 2.5, 5.0 and 7.5 mg/kg animal), diluted in 50 μL of Phosphate-Buffered Saline (PBS) by intraperitonial injection. The groups were separated according to different concentrations of the enzyme to determine the minimum dose capable of rendering the animals' blood incoagulable. Animals inoculated with PBS were used as negative control. After 3 hours, the animals were anesthetized by intraperitonial injection of a combination of ketamine (35 mg/kg) and xylazine (7 mg/kg) and the blood was depleted by cardiac puncture in the absence of anticoagulant. Then, the collected blood was immediately deposited in a glass tube and the time taken to form a visible fibrin clot was measured.

### Determination of prothrombin time (PT), activated partial thromboplastin time (APTT) and plasma fibrinogen concentration

For determining the blood coagulation parameters, male Swiss mice (18-22 g), divided into groups of 3 animals each, were intraperitoneally inoculated with rCollinein-1 at the doses of 0.5 and 1.0 mg/kg of animal, in 50 μL of PBS. Animals inoculated with PBS were used as negative control. After 3 hours of inoculation, mice were anesthetized as described above, and blood was collected by cardiac puncture using 3.2% sodium citrate as anticoagulant in a ratio of 1/10 (v/v). The collected blood was centrifuged at 1500 *xg* for 15 min at room temperature and the plasma was used to determine the PT, APTT and plasma fibrinogen concentration using commercial kits (Wiener Laboratory SAIC, CABA - AR), according to the manufacturer’s recommendations.

### Analysis of plasma proteins

Male swiss mice (18-22 g) were treated with intraperitoneal injection of rCollinein-1 in 50 μL PBS (1 mg/kg). Control animals were treated with the same volume of PBS, without the enzyme. After 3 h of injection, animals were euthanized as described above, and the blood was collected by cardiac puncture in the presence of sodium citrate (3.2%). Prior to treatment, an aliquot of blood was collected from each animal by a small incision in the tail and the sample was used as the 0 h time. Plasma samples were separated and applied (0.4 μL) on the agarose gel, which was then stained with black starch.

### Statistical analysis

Analysis of statistical variance (ANOVA) and the Tukey test, with a significance of 5% (p <0.05), were performed using GraphPad Prism software, version 5.0 (GraphPad Software Inc., San Diego, CA - USA).

## Results

### Protein production and purification

Heterologous expression of rCollinein-1 was previously standardized in minimal medium (BMM), pH 7.0, as described by Boldrini-França [40]. Expression of rCollinein-1 in minimal medium results in the cleavage of its 6x-His tag, precluding its purification from the culture medium by immobilized metal affinity chromatography (IMAC). Thus, in this work, rCollinein-1 was expressed in a medium with complex supplementation (BMMY - 1% yeast extract, 2% peptone, 1.34% YNB, 4 x 10^-5^ M biotin, 1% methanol, 100 mM potassium phosphate buffer, pH 6.0) to prevent the protein from undergoing proteolytic processing.

**Figure 1:**
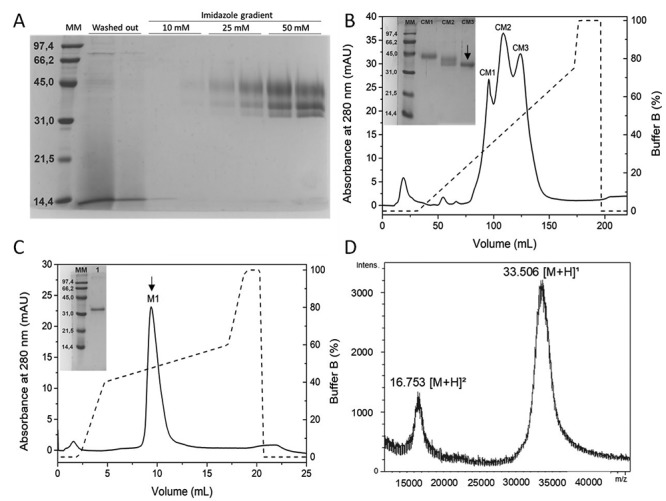
**Expression and purification of rCollinein-1**. A: 13,5% SDS-PAGE of fractions eluted from the first purification step of rCollinein-1 on a Ni^2+^-Agarose column. Fractions were eluted with a segmented gradient from 10 mM to 250 mM imidazole. MM, molecular marker; washed out, proteins eluted from the column with buffer without imidazole. The recombinant protein is indicated by the arrow. Fractions eluted with 25 and 50 mM imidazole were collected and submitted to a second purification step. B: Second purification step on an ion exchange column (CMC52). Elution was performed in a segmented gradient of sodium acetate buffer, pH 5.0 (50 mM to 1 M). The fractions (CM1, CM2 and CM3) were analyzed by 13.5% SDS-PAGE (inserted panel). C: Chromatographic profile of fraction CM3 on ion exchange chromatography (Mini S^®^ column), using the same buffer described for CMC52. Insert: 13,5% SDS-PAGE of fraction M1. D: MALDI-TOF analysis of fraction M1.

The first chromatographic step by IMAC was carried out with the culture medium previously centrifuged and filtered. The fraction containing the recombinant collinein-1 was eluted from the column with 25 and 50 mM imidazole (Fig. 1A). When analyzed by SDS-PAGE, the fraction presented three coeluted protein bands, which were then submitted to a second chromatographic step in a weak cation exchange column (CMC52). The ion exchange in CMC52 resulted in an efficient separation of these three components (Fig. 1B), which were identified by N-terminal sequencing. Two bands presenting molecular mass around 30 kDa were identified as rCollinein-1 (data not shown), indicating that the recombinant protein was present in the medium in two different populations. The band with higher molecular mass was separated from its contaminants in the second chromatographic step (fraction CM3, Fig. 1B), and its purity was confirmed by strong ion exchange chromatography in a MiniS column (Fig. 1C) and MS analysis (Fig. 1D). So, modifications in the purification protocol resulted in the expression of a soluble recombinant protein of 33.5 kDa, with an intact C-terminal, containing the 6x-His tag that allowed its purification using an IMAC as the first chromatographic step.

### Functional characterization

**Figure 2:**
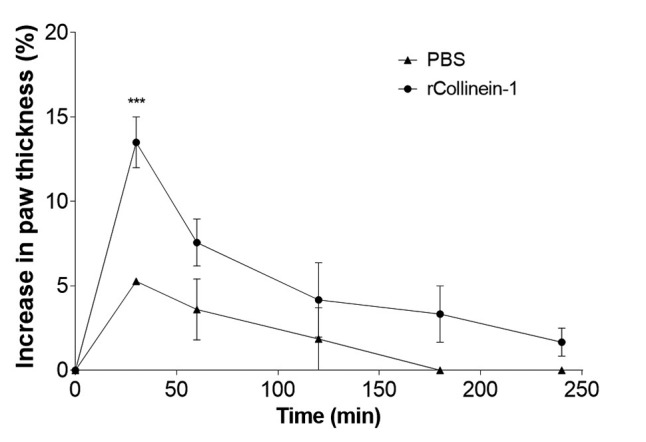
**Edematogenic activity of rCollinein-1**. Male Swiss mice were divided into two groups of four animals each. One group was treated with rCollinein-1 (10 μg diluted in 50 μl PBS) by subplantar injection in the left hind paw. The other group was treated with PBS as a negative control. Paw thickness was measured with a low-pressure plethysmometer. Data were expressed as means ±S.E.M. from four separated experiments. ^***^ *p* <0.001 compared to the negative control.

**Figure 3:**
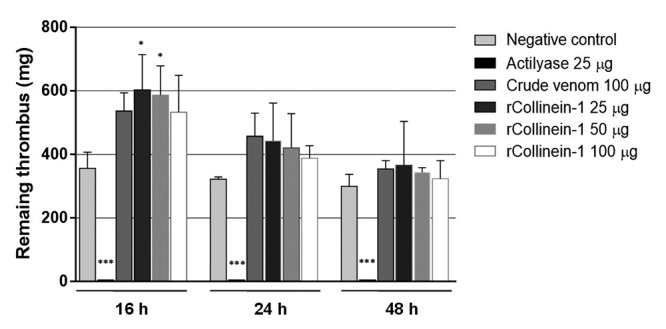
**Thrombolytic activity of rCollinein-1**. Thrombolytic activity was assessed on blood clots formed *in vitro*, as described by Toni [41]. For this, 500 μL of fresh blood (collected from healthy volunteers without anticoagulant addition) was added to 24-well plates, followed by incubation for 1 h at room temperature for clot formation. After this period, the clots were incubated for 16, 24 and 48 hours at 37 °C with different concentrations of the enzyme (25, 50, 100 μg in 500 μL of saline). Samples containing only saline and 25 μg Actilyase were used as negative and positive controls, respectively. Crude venom was also tested at a concentration of 100 μg/μL. After incubation, thrombolytic activity was estimated based on the weight of the remaining wet clot. Data were expressed as means ±S.E.M. from three separated experiments. ^*^ *p* < 0.05 and ^***^ *p* < 0.001 compared to the negative control.

The mouse paw edema induced by recombinant collinein-1 was evaluated. The enzyme induced a more prominent paw thickness increase after 30 min of the injection, although the induced edema did not represent an increase greater than 15% in the paw thickness (Fig. 2). The toxin was not able to degrade blood clots *in vitro* at any of the tested doses, even after 48 h of incubation (Fig. 3). In the thrombolytic assay, it was possible to observe an increase of the clot weight after 16 h of incubation, which can be explained by the formation of a loose clot, induced by rCollinein-1, with the remaining fibrinogen in the blood sample, which was progressively dissolved during the incubation time.

**Figure 4:**
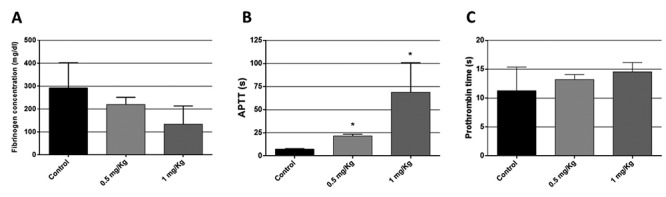
**Effect of rCollinein-1 on coagulation parameters**. For analysis of blood coagulation parameters, male Swiss mice (18 - 22 g) groups (n=4) were inoculated intraperitoneally with different doses of rCollinein-1. Animals inoculated with PBS were used as control. After 3 hours, the animals were euthanized and the blood was collected by cardiac puncture, using sodium citrate as anticoagulant (50 μL/mL). The collected blood was centrifuged, and the plasma was used to determine the concentration of fibrinogen (a), activated partial thromboplastin times (b) and prothrombin time (c). * *p* < 0.05 compared to the negative control.

rCollinein-1 led to fibrinogen depletion when injected intraperitoneally in mice, resulting in the blood incoagulability at a dose of 7.5 mg/kg. To investigate the effects of rCollinein-1 in some coagulation parameters, the prothrombin time, activated partial thromboplastin and plasma concentration of fibrinogen were determined in mice treated with the recombinant toxin. Treatment with rCollinein-1 led to an increase in the activated partial thromboplastin time at the dose of 1 mg/kg, while the other parameters, such as fibrinogen concentration and prothrombin time were not significantly altered in the tested doses (Fig. 4B).

**Figure 5:**
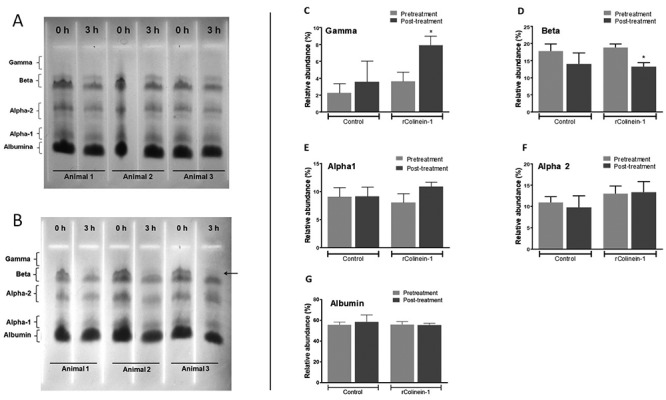
**Effect of rCollinein-1 on plasma protein**. Plasma proteins of animals not treated (a) and treated (b) with rCollinein-1 (1 mg/kg) were evaluated by agarose gel electrophoresis. After 3 h of intraperitoneal injection, the animals were anesthetized, and the blood was collected by cardiac puncture in the presence of sodium citrate. Prior to treatment, an aliquot of blood was collected from each animal by a small incision in the tail and the sample was used as 0 h. The collected blood was centrifuged at 500 x*g*, the plasma (0.4 μL) was applied on an agarose gel, which was then stained with black starch. The arrow indicates the fade of the first band of beta-globulin zone. Relative abundances of Gamma (c), Beta (d), Alpha-1 (e), Alpha-2 (f) and albumin (g) zones were determined by densitometry of the electrophoretic bands. * *p* < 0.05 compared to pretreatment condition.

Plasma of animals treated with rCollinein-1 were analyzed by agarose gel electrophoresis to evaluate the effect of the toxin on plasma proteins. rCollinein-1 significantly increased the intensity of the gamma zone, which presented a diffuse intensification of the gamma-globulin fraction (Fig. 5C). Another zone significantly altered after rCollinein-1 treatment is the beta-zone, which correspond to β-globulins (Figures 5D), indicating that the recombinant enzyme may act on the consumption of the components of this zone. The other plasma protein zones were not significantly altered by the toxin.

## Discussion

The development of biotechnological processes of recombinant protein production using the methylotrophic yeast *P. pastoris* has become one of the best low-coast strategies to obtain glycoproteins with correct folding [42, 43]. Several scientific works report that *P. pastoris* system can be used for production of proteins with potential biotechnological and therapeutic applications [15, 44-48]. This heterologous system can be easily manipulated regarding its genetic material, and can reach high cell density in culture media, which favors the large-scale production of recombinant proteins [49].

Collinein-1 is a highly thermostable thrombin-like serine protease from *C. d. collilineatus* venom that induce blood clots formation by releasing fibrinopeptides A and B from fibrinogen. The expression of recombinant collinein-1 (rCollinein-1) with *in vitro* functional integrity in *P. pastoris* system was previously reported by our group [40]; however, the previously obtained protein presented low solubility and lack of the 6x-His tag, possibly due to a proteolytic processing, which impaired its purification. In the former reported protocol, the expression was performed in minimal culture medium (BMM medium: 1.34% YNB, 4 x 10^-5^ M biotin, 1% methanol, 100 mM potassium phosphate buffer, pH 7.0), without nutritional supplementation. Thus, in the present work, we report an alternative standardized protocol for rCollinein-1 production, based on the previously reported strategy but with some modifications, such as the protein expression in complex culture medium (BMMY medium: 1% yeast extract, 2% peptone, 1.34% YNB, 4 x 10^-5^ M biotin, 1% methanol, 100 mM potassium phosphate buffer, pH 6.0).

In this new protocol, the protein was produced with structural integrity, presenting the intact 6x-His tag, which allowed the establishment of a simplified purification process by using immobilized metal ion affinity chromatography (IMAC) as the first chromatographic step, followed by the purification refinement in an ion-exchange column. The immobilized metal ion affinity chromatography is the first choice in the purification of recombinant proteins containing poly-histidine tags [50]. This methodology is based on the interaction of the imidazole ring of histidine residues present in the N or C-terminal of proteins with the immobilized nickel in the stationary phase of the column.

The fraction containing rCollinein-1 was eluted from IMAC with 25 to 50 mM imidazole and presented three protein bands when analyzed by SDS-PAGE, which were separated by cation exchange in a CMC-52 column. These three coeluted proteins underwent amino-terminal sequencing, revealing that the two bands of lower molecular mass corresponded to rCollinein-1 (data not shown). This result indicates that recombinant collinein-1 may be present in the media in two forms with different carbohydrate content. SVSPs are usually glycoproteins presenting different proportions of N- and O-linked glycosylations in non-homologous positions, which can lead to variation in their molecular mass up to 40 kDa [7].

Collinein-1 has one putative N-glycosylation site (N-X-S) in a conserved position when compared to other homologous SVSPs [40]. The N-glycosylation process is initiated by the transfer of an oligosaccharide (Glc3Man9GlcNAc2) to an asparagine residue in the N-X-S/T consensus sequence [51]. Then mannose residues can be incorporated into this nucleus, extending the carbohydrate structure [52]. It is known that the exacerbated induction of heterologous protein expression can cause a stress on the endoplasmic reticulum of the yeast, leading to variations in recombinant protein processing [53, 54], which can explain the presence of these two rCollinein-1 populations with different molecular masses. As only the population of higher molecular mass could be obtained with satisfactory purity, as confirmed by the chromatography in MiniS column and MS analysis, this population was chosen for further characterization of the recombinant protein.

The final yield of rCollinein-1 is approximately 13.2 mg of soluble protein per liter of culture medium. Although the yield of recombinant collinein-1 reported here is lower than that reported in the expression using minimal medium (56 mg/L of culture medium), the problem related to protein solubility was solved and protein recovery was enhanced.

The previously reported recombinant collinein-1 presented *in vitro* functional integrity when compared to its native form. However, *in vivo* properties of rCollinein-1, as well as its ability to activate the fibrinolytic system, had not been evaluated thus far. In this context, the recombinant protein was characterized regarding its effects on edema induction, coagulation parameters, plasma proteins and fibrinolytic activity.

The recombinant collinein-1 was able to induce a discreet paw edema in mice 30 min after injection, leading to an increase in paw thickness of less than 15%. Some serine proteases have been previously tested for induction of paw edema in mice, such as BpirSP41 and BpirSP27 from *Bothrops pirajai* [55], BpSP-I from *B. pauloensis* [56], TLBm from *B. marajoensis* [57] and Cdtsp 2 from *C. d. durissus* [58], in which all of them induced mild to moderate edema formation. The edema induced by snake venoms depends mainly on the release of pro-inflammatory mediators, such as arachidonic acid metabolites (prostaglandins and leukotrienes), lipoxygenase products, histamines, serotonin and nitric oxide [59, 60]. Cdtsp 2 induces mild paw edema in murine models by degrading protease-activated receptors PAR1 and PAR2, which lead to activation of phospholipase C (PLC) and protein kinase C (PKC) to mobilize arachidonic acid, while inducing oxidative stress [58].

Although presenting proinflammatory toxins, accidents caused by *Crotalus* snakes induce little or no local effect [61]. Some studies have shown that *C. d. terrificus* venom can lead to a down-regulation in the humoral and cellular immune response [62, 63] and crotoxin, that is the main toxin in the venom, presents long-lasting anti-inflammatory properties, affecting immune cell activity and migration [63-66].

rCollinein-1 causes a fibrinogen depleting effect when injected intraperitoneally in mice, causing blood incoagulability at the dose of 7.5 m/kg. The recombinant toxin was also able to enhance the activated partial thromboplastin time (APTT) in mice at the dose of 1 mg/kg but did not significantly affect the other assayed coagulation parameters. The increase in APTT may be correlated with the decrease in plasma fibrinogen concentration, although the enzyme may also be acting on other points of coagulation, like factors XII, XI, IX and VIII of the intrinsic pathway or factors X, V and prothrombin of the extrinsic pathway [67].

The physiological conversion of fibrinogen into fibrin is catalyzed by thrombin, which promotes the cleavage of the N-terminal portions of the Aα and Bβ chains on the fibrinogen E-nodule, releasing the fibrinopeptides A and B, respectively. Removal of fibrinopeptides exposes the polymerization sites in the N-terminal of α and β chains, which interact with the polymerization sites in the C-terminal of the same chains, resulting in insoluble fibers, named fibrin monomers. Thrombin also activates coagulation factor XIII, which is a transglutaminase that catalyzes the formation of isopeptide bonds between fibrin γ chains, leading to the formation of γ-γ dimers, and between α chains of several fibrin molecules, forming α-polymers. The fibrin clot stabilized by these cross-links is resistant to the action of the fibrinolytic system, forming a tight blood clot [68].

SVTLEs present a similar activity to that of thrombin, promoting the release of fibrinogen A and/or B from fibrinogen α and β chains, respectively. However, most thrombin-like enzymes differ from thrombin by lacking the ability to activate coagulation factor XIII, leading to the formation of loose clots that are easily removed by the fibrinolytic system. The result is a hypofibrinogenemia condition with consequent blood incoagulability [69].

Although SVSPs act primarily on converting fibrinogen into fibrin monomers, some of these enzymes may also have direct fibrinolytic effect or may activate the fibrinolytic system either in direct or indirect ways [2, 70]. The serine protease ACC-C from *Agkistrodon contortrix contortrix* promotes protein C activation, leading to factor Va degradation [71]. TSV-PA from *Trimeresurus Stejnegeri* [72], Haly-PA from *A. halys brevicaudus* [73] and LV-PA from *Lachesis muta muta* [74] are serine proteases that, like u-PA and t-PA, convert plasminogen into plasmin, activating fibrinolysis. SVSPs can also present direct fibrinolytic activity, degrading α and/or β chain of fibrin, such as the BpirSP27 and BpirSP41 of *B. pirajai* [55] and harobin from *Lapemir hardwickii* [75]. rCollinein-1 did not degrade blood clot in none of the tested dose, being unable to directly or indirectly activate the fibrinolytic system.

The effect of rCollinein-1 on plasma proteins was tested by analyzing the plasma of treated mice on agarose gel electrophoresis. Electrophoresis is an efficient and widely used method to determine plasma or serum protein components. Plasma protein bands include albumin, alpha1, alpha2, beta and gamma globulins. The plasma proteins comprise enzymes, hormones, antibodies, proteins responsible for the maintenance of osmotic pressure, among other components. Albumin is the major protein component of plasma, comprising 35-50% of total proteins, and plays a key role in the transport of various endogenous and exogenous substances. Among globulins, alpha1 and alpha2 globulin zones in agarose electrophoresis include many of the acute phase proteins (e.g. alpha2, macroglobulin and haptoglobin), which are indicative of acute inflammatory processes. The beta zone comprises transferrin, the complement component 3 (C3), and several proteins involved in blood coagulation. Finally, the gamma zone includes immunoglobulins (IgA, IgM, IgE and IgG), although the density of this band is mainly due to IgG [76-78].

The recombinant collinein-1 leads to a decrease in the intensity of β-zone, indicating that rCollinein-1 degrades some of its components, which comprises coagulation factors. rCollinein-1 may also have cleaved C3 in this zone, since some SVSPs are known to act upon this component, such as flavoxobin from *Trimeresurus flavoviridis*, activating the complement alternative pathway [79]. Moreover, two thrombin-like enzymes from *B. pirajai* modulate the complement system, although it is not possible to predict if this effect is due to activation or inactivation of the complement components or a result of blocking the activation/modulation pathways of this system [55]. Another zone that had its intensity affected by rCollinein-1 is the gamma zone, which presented a diffuse increase of the gamma-globulin fraction. Increase in immunoglobulins expression are generally indicative of some pathologic conditions, such as liver diseases, chronic infections, metastatic carcinoma and cystic fibrosis [76], although none of these conditions can be directly related to the previously known physiological effects induced by SVTLEs.

## Conclusions

In the present work, the recombinant protein was expressed based on the previously reported protocol, with some modifications, resulting in the production of a recombinant collinein-1 with improved solubility and structural integrity. rCollinein-1 was not able to dissolve blood clots and did not induce expressive paw edema. On the other hand, this enzyme leads to blood incoagulability and increased activated partial thromboplastin time in mice. These results indicate that rCollinein-1 has potential application as a fibrinogen depleting drug to prevent thrombus formation in some pathologies and medical procedures, in certain diagnostic assays or may even serve as tools for studies related to hemostasis. Even though the therapeutic potential of rCollinein-1 is evident, the main bottleneck in bioprospecting SVSPs is their limitation in large-scale production from natural sources. Thus, the improved biotechnological strategy in producing the recombinant collinein-1 may represent a step forward in applying this toxin as a biopharmaceutical.

## Abbreviations

 Not applicable.
